# *In Silico* identification and annotation of non-coding RNAs by RNA-seq and *De Novo* assembly of the transcriptome of Tomato Fruits

**DOI:** 10.1371/journal.pone.0171504

**Published:** 2017-02-10

**Authors:** Daria Scarano, Rosa Rao, Giandomenico Corrado

**Affiliations:** Dipartimento di Agraria, Università degli Studi di Napoli “Federico II”, via Università 100, Portici (NA), Italy; University of Western Sydney, AUSTRALIA

## Abstract

The complexity of the tomato (*Solanum lycopersicum*) transcriptome has not yet been fully elucidated. To gain insights into the diversity and features of coding and non-coding RNA molecules of tomato fruits, we generated strand-specific libraries from berries of two tomato cultivars grown in two open-field conditions with different soil type. Following high-throughput Illumina RNA-sequencing (RNA-seq), more than 90% of the reads (over one billion, derived from twelve dataset) were aligned to the tomato reference genome. We report a comprehensive analysis of the transcriptome, improved with 39,095 transcripts, which reveals previously unannotated novel transcripts, natural antisense transcripts, long non-coding RNAs and alternative splicing variants. In addition, we investigated the sequence variants between the cultivars under investigation to highlight their genetic difference. Our strand-specific analysis allowed us to expand the current tomato transcriptome annotation and it is the first to reveal the complexity of the poly-adenylated RNA world in tomato. Moreover, our work demonstrates the usefulness of strand specific RNA-seq approach for the transcriptome-based genome annotation and provides a resource valuable for further functional studies.

## Introduction

Next Generation Sequencing (NGS) technologies are having an important impact on genomic research because they can be employed to address questions unapproachable with earlier tools. The power and speed of NGS allow to generate high-quality genome sequences [1, 2], compare genomes across multiple samples [3, 4], map structural variations [5–7] and identify polymorphisms [8, 9].

Compared to other existing methods, Whole Transcriptome Shotgun Sequencing, better known as RNA-seq, has provided a very powerful alternative to gene expression analysis [10, 11]. High-throughput sequencing-based approaches of the RNA world are also used to assemble, improve or extend the transcriptome of an organism, either by reference-based or de novo strategies [12–16]. They also allow a comprehensive discovery of novel genes and transcripts at a fraction of the cost and time of the conventional sequencing methods [17, 18]. Moreover, NGS technologies can reveal previously hidden dimensions in transcriptomes, such as isoforms, novel splice junctions, long non-coding RNAs and expression of antisense RNAs [14, 15, 19–22]. Therefore, with the recent increased availability of plant genome sequences, RNA-seq is being also employed to improve and expand existing genome annotations [18, 23–25]. Finally, considering the size of plant genomes, RNA-seq is also a cost-effective strategy to reveal sequence variations in coding sequences [25–28].

Tomato is arguably the most important vegetable in the world [29]. Cultivation is largely based on hybrid varieties, although in certain areas, local accessions fetch a premium price. The importance of tomato cultivation is increasing worldwide also due to growing attention of the potential health benefits of the compounds of berries. Moreover, in recent years, tomato is becoming a widely used research model organism for fleshy fruit biology, as well as in relation to fruit size and shape evolution, plant stress response, domestication and adaptation [29].

The complete tomato genome sequence was released in 2012 [30]. The variety chosen was ‘Heinz 1706’, a processing inbred cultivar. The genome size is approximately 930 Mb and of the 34,725 protein-coding genes, 30,855 are supported by RNA-seq data. The official functional annotation for the tomato genome, provided by the International Tomato Annotation Group (ITAG), reports 26,026 genes that are associated to Gene Ontology (GO) terms describing their functions, for a total of 2,108 unique GO terms. All this information is a precious resource especially for functional studies [31].

Considering the recent development of plant genomics, transcriptomic studies relies on reference annotation that not necessarily capture the full catalogue of transcripts and their variations [32]. With the aim to provide information useful to increase our understanding of the tomato fruit, the focus of the present study was to present an extended transcript catalogue of this organ. To this goal, we obtained 12 sets of strand-specific RNA-seq data from tomato fruits of two varieties in two environments. Starting from a genome-guided assembly, we report a comprehensive analysis of the tomato transcriptome, improved with a collection of 39,095 transcripts that include splicing variants, transcripts overlapping annotated loci, natural antisense transcripts and transcripts completely absent from the official tomato annotation. Furthermore, we investigated the genetic diversity by analyzing the polymorphisms in the varieties under investigation.

## Materials and methods

### Plant material

Transcriptomic analysis was carried out on berries of two tomato (*Solanum lycopersicum* L.) cultivars, ‘Kiros’ (K) and ‘Docet’ (D). ‘Kiros’ is an inbred variety of local origin, while ‘Docet’ is a hybrid cultivar (Seminis). Both varieties are used for tomato processing. Plants were grown in two different locations near Naples (Italy), Acerra (AC) and Brusciano (BR), with different soil types, volcanic, defined as Typic Haplustoll, and alluvial, defined as Typic Calciustepts, respectively [33], to have a richer catalogue of expressed molecules. Plants were grown in the traditional spring to summer crop cycle at the experimental fields of the European Environmental Company (EURECO), Acerra (Italy), which also provided the seeds. The same day, fully ripe berries from three different plants, representing biological replicates, were collected. For each sample, three fruits from the same plants were pooled in order to reduce biological variation. Fruits were immediately frozen in liquid nitrogen and stored at -80°C until RNA isolation.

#### RNA isolation and evaluation

Samples were ground to a fine powder with liquid nitrogen using a sterile mortar and pestle. RNA isolation, from around 300 mg of powdered frozen tomato fruits, was performed by a phenol/chloroform procedure followed by a lithium chloride precipitation, as described [34]. To reduce technical variation, for each replicate, total RNA of each sample was pooled from 8–12 isolations. To get rid of possible DNA contamination, 10 μg of total RNA were treated with 6 U of DNAse I Amplification Grade (Invitrogen) in 1X DNAse I Reaction Buffer (Invitrogen). DNase I was removed by phenol/chloroform purification followed by RNA precipitation through the addition of 0.1 volume of 3M Sodium Acetate (pH 7.0) and three volumes of ethanol. The integrity of RNA molecules was evaluated by electrophoretic RNA measurements, recorded with an Agilent 2100 Bioanalyzer. Only samples with high (>8) and similar (overall s.d. across all samples: ± 0.21) RNA Integrity Number were further processed.

### Library construction

We converted the mRNA of total RNA into a library of template molecules of known strand origin using the reagents provided in the TruSeq Stranded mRNA Sample Preparation Kit (Illumina). Briefly, the poly-A mRNAs were purified from 8 μg of total RNA using poly-T oligos attached to magnetic beads. Following purification, the mRNA was fragmented into small pieces using divalent cations under elevated temperature. The cleaved RNA fragments were copied into first strand cDNA using reverse transcriptase and random primers. Strand specificity was achieved by using dUTP (instead of dTTP) in the second strand cDNA synthesis, using DNA Polymerase I and RNase H. After 3’ end adenylation and adapters ligation, the products were purified and selectively enriched by PCR to create the final ds cDNA library. Libraries were validated and quantified using an Agilent 2100 Bioanalyzer (Agilent Technologies), and pooled such that each index-tagged sample was present in equimolar amounts, with a 2 nM final concentration of the pooled samples.

### RNA-sequencing and data analysis

The pooled samples were subject to cluster generation and sequencing using a HiSeq 1500 System (Illumina) in a 2x100 paired-end format at a final concentration of 8 pmol. In total, 12 libraries were sequenced at the Laboratory of Molecular Medicine and Genomics, Department of Medicine and Surgery (University of Salerno), according to manufacturer’s instructions. Quality control on raw sequences was carried out with FastQC (www.bioinformatics.babraham.ac.uk/projects/fastqc/). Prior to further analysis, we removed low quality portions while preserving the longest high quality part of NGS reads, using Trimmomatic [35]. To increase quality and reliability, the minimum length allowed was 25 bp and the minimum quality score was set to 35. Briefly, the RNA-seq analysis was a two-stages process that involved reads processing and assembly of new transcripts, and annotation of lncRNAs and sORFs. The first step included the alignment of the reads against the reference genome, the reference‐guided assembly of new transcripts, the annotation and classification of new loci. The second step consisted in the assembly and annotation of lncRNAs and sORFs by using specific pipelines [36]. Transcripts encoding a protein shorter than 200 amino acids were BLASTed against the NCBI NR database. Sequences having a match with a known protein (e-value lower than 0.001) were annotated as sORFs.

The TopHat script v.2.0.11 [37] was used to align high-quality RNA-seq reads to the tomato genome (version SL2.50). The alignment files were filtered to remove duplicated reads and those with a low mapping quality (<30). Data were then used as input for Cufflinks (v. 2.2.1) [15] together with the ITAG2.40 annotation to integrate the official assembled transcripts with the new ones. In-house pipelines and manual curation were performed to remove possible annotation errors. Specifically, erroneous gene fusions and loci completely overlapping with other loci in the same strand orientation were removed.

Functional annotation of sequences and data mining on the resulting annotations, primarily based on the GO vocabulary was performed with Blast2GO with manual curation [38]. This also allowed to obtained information expressed as a controlled vocabulary of functional attributes via the GO. Gene-annotation enrichment analysis was performed employing the Fisher’s exact text corrected for multiple testing (p<0.05). Summarization and visualization of enriched GO terms was carried out using REVIGO [39].

### Variant calling and annotation

The Simply Unified Pair-End Read (S.U.P.E.R.) workflow was used to identify sequence variations [40]. Briefly, the pipeline removes adaptors, performs trimming, checks data quality, pairs existent reads in both files (forward and reverse reads), aligns sequences on reference genomes and call sequence variations. S.U.P.E.R output were filtered to extract significant variations (high confidence calls) affecting the samples (quality score higher than 30 and total depth for each variant higher than 6). The assignment of functional information to DNA variants was performed with SNPeff (v 3.6) [41]. We categorized each variant based on its relationship to coding sequences in the genome and on how the variant may change the coding sequence and affect its gene product.

## Results

### Mapping of RNA-seq reads to the tomato reference genome

The current version of the tomato reference genome (SL2.50) was released in 2014. The estimated genome size is 930 Mbp and 84% is annotated (782 Mbp). The structural annotation comprises 34,725 genes models, with a total of 160,007 exons and 125,280 introns.

The 12 RNA-seq dataset from tomato fruits (2 varieties, 2 environments, 3 biological replicates) were mapped against the reference genome. The mean input and its standard deviation was 104,487,055.8 ± 5,795,556.7 reads and 102,660,154.5± 5,854,766.6 reads per sample, before and after the data quality control respectively, for a total of 1,253,844,670 and 1,231,921,854 reads (Table 1). The mean mapping rate was 90.7 ± 2.9%, which indicated that a small portion of reads were not counted in the RNA-seq read mapping process (Table 1).

**Table 1:** RNA-seq main metrics. The summary statistics, calculated against the current version of tomato reference genome, report the number of reads before and after the quality control, the number of high quality mapped reads and the percentage of mapped reads.

Sample Name	Reads before data quality control	Reads after data quality control	Mapped reads (quality >30)	Percentage of mapped reads (quality >30)
K1AC	107,052,898	105,145,278	89,432,957	85.06
K2AC	98,134,344	95,213,184	87,423,415	91.82
K3AC	101,499,130	99,864,816	90,816,835	90.94
K1BR	103,309,678	101,602,468	94,521,908	93.03
K2BR	98,891,860	97,159,942	87,799,190	90.37
K3BR	97,227,964	95,562,146	86,827,366	90.86
D1AC	106,151,188	104,670,078	88,918,296	84.95
D2AC	110,629,588	108,820,606	99,896,289	91.80
D3AC	113,276,110	111,352,484	102,227,365	91.81
D1BR	113,820,186	111,986,058	102,172,834	91.24
D2BR	104,189,040	102,501,582	95,841,445	93.50
D3BR	99,662,684	98,043,212	91,691,833	93.52
Total	1,253,844,670	1,231,921,854	1,117,569,733	
Mean	104,487,056.8	102,660,154.5	93,130,811.1	90.07
s.d.	5,795,556.7	5,854,766.6	5,713,925.4	2.92

Legend: K: Kiros variety; D: Docet variety; AC: Acerra location; BR: Brusciano location.

### Improvement of the tomato annotation

To extend the annotation of the tomato genome, the 12 RNA datasets were used to uncover novel transcripts. The high quality reads were aligned to the *Solanum lycopersicum* reference genome sequence (SL2.50). The resulting alignments were filtered to remove duplicated reads and those with a low mapping quality. The data were then used, together with the ITAG2.40 annotation file, to assemble transcripts. We found in total 73,820 transcripts and 34,725 matched those in the available reference. The new transcripts (39,095) were grouped into four classes.

Class J includes transcripts that are new splicing variants of already annotated loci; class O consists of transcripts that overlap partially with already annotated loci and class X comprises new transcripts that have an antisense orientation to already annotated loci. Finally, we indicate as TCONS new transcripts that are absent from the available annotation.

**Fig 1:**
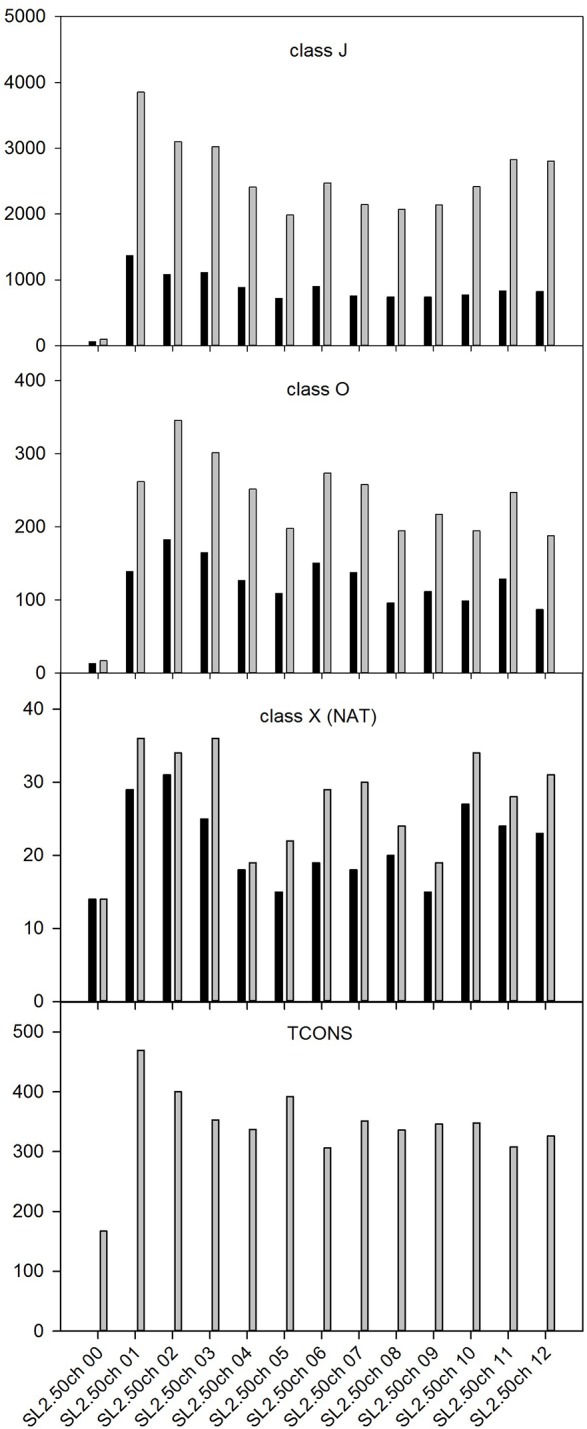
Number of transcripts (grey bars) and their corresponding genes (dark bars) per chromosome for each class of transcripts. J: new splicing variants; O: transcripts with an overlap with already annotated loci; X: transcripts that have an antisense orientation to already annotated loci; TCONS: new transcripts that are new and absent from the available official annotation.

The Class J included 31,349 splicing variants corresponding to 10,788 unique tomato annotated genes. We found from a minimum of 1 to a maximum of 24 splicing variants per gene, for an average of 2.91 variants per gene. The distribution of splicing variants transcripts and corresponding genes per chromosome is reported in Fig 1. The average number of splicing variant transcripts per chromosome is 2,41. The highest number of genes with splicing variants (1,37) was relative to the SL2.50ch01 chromosome.

The analysis of RNA-seq reads revealed 2,951 transcripts that partially overlap with already annotated loci (class O), corresponding to 1,548 tomato genes distributed in all tomato chromosomes. On average, we found 227 class O transcripts per chromosome (Fig 1). Moreover, the analysis indicated the presence of 356 new transcripts that are antisense to already annotated loci (Class X). These transcripts, which can be also defined Natural Antisense Transcripts (NATs), refer to 278 genes, with an average of 27.38 transcripts per chromosome (Fig 1). The classification of NAT pairs based on their overlapping pattern is presented in Table 2.

**Table 2:** NAT transcripts classification based on their overlapping patterns. Contained: overlap involving coding exons; tail-to-tail: overlap involving 3' regions and coding exons; head- to-head: overlap involving 5' regions and coding exons; fully overlapping: involving the entire NAT sequence. *cis*-NATs: transcribed from opposing DNA strands at the same genomic locus; *trans*-NATs are transcribed from separate loci.

NAT orientation	N°	NAT classification	
		*cis*	*trans*
contained	487	112	375
tail-to-tail	157	99	58
head-to-head	100	53	47
fully overlapping	7	4	3

Lastly, the TCONS class includes 4439 new tomato transcripts, for an average of 341.46 transcripts per chromosome (Fig 1). The majority of the new transcripts (3,514) were non-coding. Overall, with the exception of the unplaced scaffolds (SL2.50ch00), we did not observe a chromosome specific enrichment for all classes of new transcripts, suggesting that our data provides a good coverage of the whole tomato genome.

### Long non-coding RNA (lncRNA)

Following the identification of new transcripts, we developed a new annotation by merging novel and known isoforms, and maximizing overall assembly quality. Subsequently, we performed the identification of lncRNA. These are non-protein coding transcripts longer than 200 nucleotides that lack or have small Open Reading Frames (ORFs). We found 10,774 lncRNAs, in which we further distinguished putative precursors of miRNA (88 in number), rRNA (176) and tRNA (12). The 10,774 lncRNAs included also new transcripts, belonging to the above mentioned four classes. Specifically, while 3,600 transcripts were already present in the ITAG2.40, 3,514 lncRNAs were new (i.e. TCONS), 323 belongs to the class X, 879 class to the class O and 2,458 to the class J.

In addition, we identified a total of 3,800 small ORFs (sORFs) containing RNAs. These are potentially translatable sequences that consist of a string of in-frame sense codons shorter than 200 aa. sORFs identified in this study included 12 transcripts absent from the tomato annotation (TCONS class) as well as 344 belong to the class J, 70 to class O and 2 to class X. The remaining 3,372 transcripts were already present in the ITAG2.40 annotation.

### Functional annotation of the transcripts absent from the official annotation

Transcripts that are new and absent from the official annotation were annotated essentially by extracting Gene Ontology term associations. The ontology covered three domains: cellular component (C), the parts of a cell or its extracellular environment; molecular function (F), the elemental activities of a gene product at the molecular level and biological process (P), operations or sets of molecular events with a defined beginning and end, pertinent to the functioning of integrated living units: cells, tissues, organs, and organisms.

#### Class J (splicing variants)

The assembled transcripts mainly included variants of already described transcripts, due to alternative splicing in fruits. A functional enrichment analysis was carried out considering genes, in order to remove redundancy. The identification of functional classes that statistically differ between the annotated tomato genome (version SL2.50, annotation ITAG2.40) and genes that possess previously undescribed splicing variants (10,788) revealed a total of 120 GO enriched terms, under the cellular component (11), molecular function (98) and biological process (11) domains. The most represented terms were “organelle membrane” (C), “binding” (F) and “cellular response to stimulus” (P) with 37, 3,331 and 72 annotated genes, respectively (Table A in S1 File). The analysis indicated that alternative splicing in fruits is a phenomenon that involve several functions. Among them, important categories of enriched biological processes are potentially related to fruit metabolism (Table A in S1 File).

#### Class O (transcripts overlapping annotated loci)

The assessment of differences in GO term abundance between 1,548 tomato genes of the Class O transcripts and the tomato reference genome yielded a list of 17 GO terms under the “molecular function” GO domain (Table B in S1 File).

#### TCONS (novel transcripts)

The new 4,439 TCONS sequences were firstly annotated by comparing their putative translation product against the Arabidopsis proteome (TAIR10). In total, 95 TCONS found a match with 100 experimentally validated Arabidopsis proteins. The majority of TCONS sequences were classified as lncRNA (3,514 transcripts, of which 21 were similar to Arabidopsis transcripts) and sORFs (12). The remaining 839 transcripts did not yield a functional annotation when automatically compared to Arabidopsis. The functional annotation of these sequences was then performed by transferring existing functional annotation from similar sequences identified by the Blastp algorithm. The analysis indicated that 599 (71.7%) TCONS had significant returns (i.e.: an average protein similarity higher than 50% using as threshold an e-value < 10e-6) and 6 (0.7%) had an average similarity with the retrieved proteins higher than 49% (S1 Fig). Among them, 462 could be categorized in at least one of the three principal GO domains, while 143 were did not retrieve GO information. For the 462 TCONS similar to other proteins, the ontological domain cellular component (C) was less represented, with 155 GO terms, the biological process (P) was the richest domain (with 785 terms), while 293 GO terms were present in the molecular function category (Table C in S1 File). The remaining 231 (27.6%) TCONS did not retrieve a significant similarity in the non-redundant protein NCBI database.

**Fig 2:**
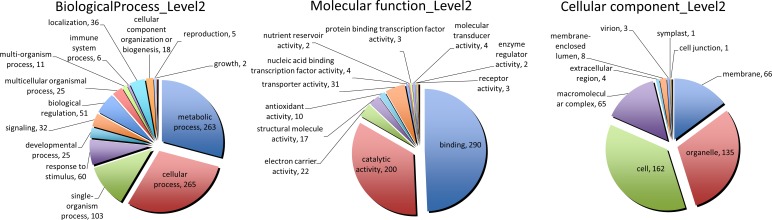
Distribution of GO terms association of the annotated class TCONS transcripts. For the three ontological domains (C: cellular component; F: molecular function; P: biological process), the pie chart reports the number of GO terms in the different categories.

Considering the level-2 distribution of the sequences in the molecular function domain, the GO term “binding” was the most abundant. “Metabolic process” and “cellular process” were the most representative GO terms analysing the biological process domain and the cellular component ontology domain is represented by “organelle” and “cell” terms (Fig 2 and Table D in S1 File). The presence of novel transcripts that are similar to known proteins implies a high value of the novel assembled transcripts.

#### Class X (NATs)

**Fig 3:**
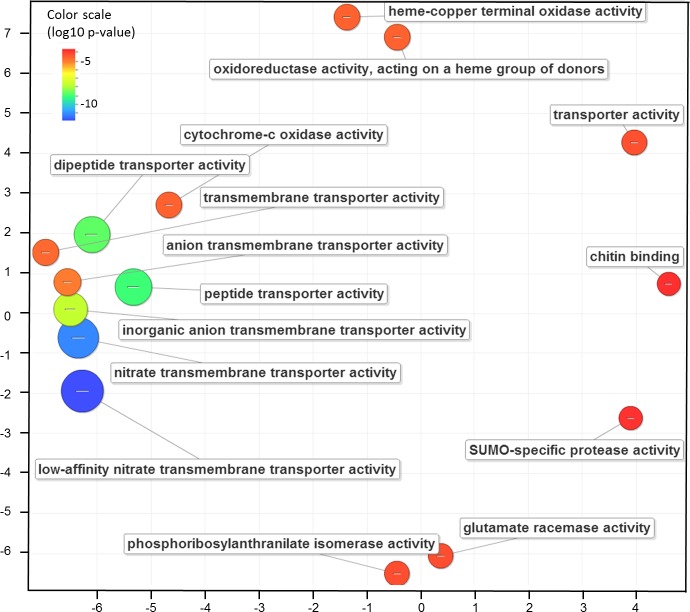
Summarization and visualization of enriched GO terms of NAT pairs in tomato fruits. The scatterplot shows the representative clusters (made of enriched GO terms of Table E in S1 File after the redundancy reduction) in a two dimensional space derived by applying multidimensional scaling to a matrix of the GO terms' semantic similarities (more semantically similar GO terms are closer). Bubble color indicates the p-value according to the legend in upper left-hand corner. Bubble size is scaled according to the frequency of the GO terms.

New Natural Antisense Transcripts of already annotated tomato loci (356; class X) were aligned to the tomato reference database for cDNA sequences (ITAG 2.40) to find complementary target RNAs. Almost all NATs (351) gave a match, for a total of 759 tomato cDNAs relative to 537 tomato genes (Table E in S1 File). A functional enrichment analysis was performed on these target genes in order to verify which are the most represented ontology domains and GO terms. The analysis revealed 43 GO terms belonging to the molecular function domain, while six were included in the biological process domain (“aromatic amino acid family biosynthetic process”, “aromatic amino acid family metabolic process”, “protein folding”, “cell wall macromolecule catabolic process”, “cell wall macromolecule metabolic process” and “cell wall organization or biogenesis”) (Table F in S1 File). We found a representative subset of the over-represented Molecular Function GO terms using semantic similarity measures. A cluster related to the directed movement of substances (organic, inorganic and ions) into, out of or within a cell, or between cells was evident in terms of semantic similarities as well as statistical significance (Fig 3). Overall, the functional analysis of the NAT pairs we identified is consistent with a significant enrichment of functions related to fruit biology.

### Variant calling

RNA-seq data were employed to discover single nucleotide variants in comparison with the tomato reference genome. The number of variants and their classification is summarized in Table 3. SNPs accounted for around 90% of total sequence variation. Around 40% of the variants were common between the two varieties. The cultivar Docet had a higher number of polymorphic sites than the inbred line Kiros. Moreover, the vast majority of variants in the Docet were in heterozygosis. As expected, the majority of variants were classified as having a limited impact, that is they were related usually to non-coding regions or affecting non-coding genes. The type of effects of the variants between the two varieties did not display notable differences in percentage. The ratio between synonymous and non-synonymous variants was higher for the heterozygous variants of the Docet cultivar (1.14) and lower for the common polymorphism (0.79).

**Table 3:** Distribution and classification of sequence polymorphisms. Unique (i.e. present only in one variety) and common (i.e. present in both varieties) polymorphisms. For the hybrid cultivar Docet, polymorphisms were distinguished in heterozygous (H) and homozygous (O) variants.

	Unique Variants						Common Variants		Total	
	Kiros		Docet (H)		Docet (O)					
**Change type**										
SNP	2,538	96.2%	6,208	97.0%	1,224	96.8%	6,104	81.5%	16,074	90.3%
IN	51	1.9%	90	1.4%	24	1.9%	1,234	16.5%	1,399	7.9%
DEL	49	1.9%	105	1.6%	17	1.3%	153	2.0%	324	1.8%
Total	2,638		6,403		1,265		7,491		17,797	
**Effects**										
HIGH	10	0.2%	35	0.3%	5	0.2%	126	0.7%	176	0.4%
LOW	665	12.0%	1,960	14.5%	304	11.3%	1,457	7.9%	4,386	10.9%
MODERATE	601	10.9%	1,591	11.8%	297	11.0%	1,514	8.2%	4,003	10.0%
MODIFIER	4,257	76.9%	9,953	73.5%	2,095	77.6%	15,272	83.1%	31,577	78.7%
Total	5,533		13,539		2,701		18,369		40,142	
**Functional type**										
MISSENSE	607	49.1%	1,599	46.3%	300	51.9%	1,530	55.1%	4,036	50.2%
NONSENSE	3	0.2%	12	0.3%	1	0.2%	20	0.7%	36	0.4%
SILENT	627	50.7%	1,841	53.3%	277	47.9%	1,226	44.2%	3,971	49.4%
Total	1,237		3,452		578		2,776		8043	

**Fig 4:**
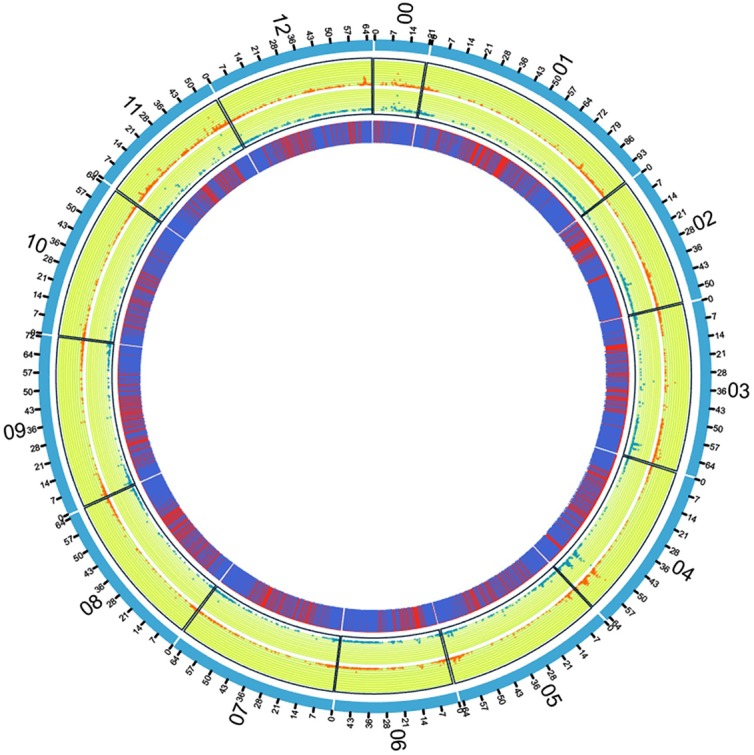
SNP distribution and density on the tomato genome in the two tomato varieties. The outer circle represents the tomato chromosomes; the inner circle graphically reports the distribution of transcribed regions (presence in blue, absence in red). The middle circles report the distribution and density of SNPs in our samples (blu dots: Docet; orange dots: Kiros). The height of each point relates to the SNP frequency in a 50 kb window.

**Fig 5:**
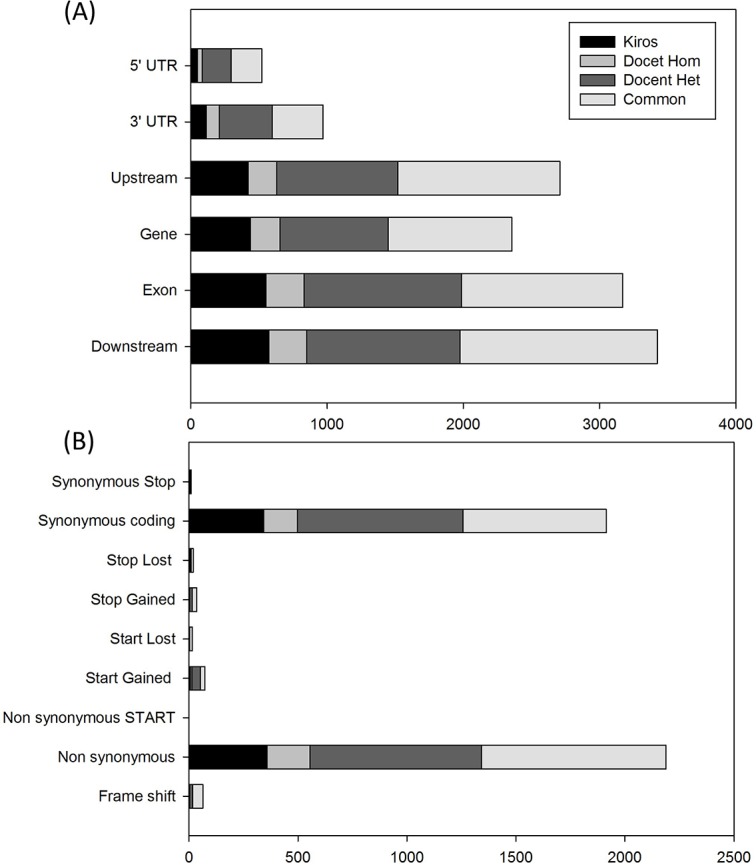
SNP classification according to their positions (A) and their effect (B).

The Kiros variety showed 1269 genes with at least one variant, while Docet had the vast majority of polymorphic genes (2404) in heterozygosis. Moreover, 3350 genes with a least one single nucleotide variation were present in both genotypes under investigation. After merging the lists of genes affected by nucleotide variation (and removing duplicates) a total of 6859 unique tomato genes had at least one variant, with an average of 527.62 polymorphic transcribed sequences per chromosome. The chromosome 4 showed the highest number of polymorphic genes (S2 Fig). Fig 4 reports the distribution and density of polymorphic sites in the two varieties. Differences in variants distribution between the two varieties were evident particularly for chromosome 11 and some regions of chromosome 3 and 12. For each gene, the variants were classified in six categories according to their positions (Fig 5A), namely downstream (if within the 1 kb region downstream from the stop codon), exon, gene, upstream (within the 1 kb region upstream from the start codon), 3' UTR and 5' UTR. Polymorphisms were also grouped into nine categories according to their effect (Fig 5B), that is frame shift, non synonymous, non synonymous start, start gained, start lost, stop gained, stop lost, synonymous coding and synonymous stop.

## Discussion

### Identification of novel transcripts

RNA-seq has provided the opportunity of generating a global view of the transcriptome of plant organs or developmental stages of scientific and economic importance. This is necessary to produce a genome-scale map that comprises both transcript structure, complexity and expression level in specific conditions [15, 42]. Accurate genome annotation, for instance, is important for a reliable gene expression quantification [43], an essential element to understand how plants grow, differentiate and respond to the environment.

In this study, we present an improved tomato transcriptome extracted from the analysis of tomato fruits using strand specific RNA-seq libraries. To limit varietal and environmental bias, we analysed two varieties grown in two experimental fields, which mainly differ in the soil type.

Through direct mapping of more than one billion of reads to the tomato reference genome, we identified 39,095 previously undescribed transcripts. In *Sorghum bicolor*, RNA-seq based gene annotations identified 34,276 new transcribed genomic regions [44]. The apple reference genome was improved by the description of 71,178 genes or transcripts, which includes 17,524 novel transcripts [23]. Moreover, in the present study the chromosomal locality of the 39,095 new transcripts was determined, denoting that they were of tomato origin.

The new transcripts were also identified from the reference-guided assembly of reads that could not be mapped initially, and were divided in four classes. Class J (new splicing variants of already annotated loci) was, as expected, the group with the higher number of transcripts. This group refers to 10,788 unique tomato annotated genes. In Arabidopsis, approximately 42% of intron-containing genes are alternatively spliced [45] and in rice, about 48% [25]. Alternative splicing variants are recognised as one of the most significant contributors to transcriptome plasticity and proteome diversity [46]. The abundance of new transcripts in the class J and their GO-annotation should reflect the fact that during ripening, fruits undergo significant biochemical, physiological and morphological changes. Considering that incomplete annotations can bias gene expression estimates [15, 42, 43], the high number of detected isoforms suggests that organ specific annotations are not only useful but also needed in tomato.

Compared with the available version of *S*. *lycopersicum* genome (SL2.50), we found 4439 transcripts that are new and completely absent from the official annotation (class TCONS). Around one hundred had a match with Arabidopsis proteins, while the majority (3,514) were classified as lncRNAs. Even if the majority of lncRNAs have been identified in animals through high-throughput transcriptome sequencing, other targeted approaches may increase this number. Although lncRNAs have been gradually recognized as crucial gene regulators also in plants, unfortunately, only few functionally characterized examples are available [47]. Twenty-one lncRNAs had a significant similarity with Arabidopsis. In this model species, the examination of different organs and conditions indicated that more than 6,000 RNAs were classified as long transcripts from intergenic regions, but analysis of fruits is not available. The limited similarities with Arabidopsis is reasonable considering the generally low conservation of lncRNA sequences among species [48]. While 12 TCONS were classified as sORFs, of the remaining 839 sequences, 72.4% putatively codes for proteins having high similarity (>49%) with other plant proteins. The majority of these putative translational products could be functionally annotated. We retrieved a variety of GO terms that covered a wide range of molecular functions and biological processes, and we did not observe a significant enrichment. The translational products that could not be functional annotated (143) were associated to uncharacterized proteins annotated as ‘unknown’ or ‘hypothetical’.

Natural Antisense Transcripts represented the less abundant class of the newly identified transcripts. It has been estimated that in plants, less than 10% of all transcripts overlap as cis-NAT, a percentage that is expected to be lower than in mammals. In the present study, by applying a strand-specific RNA-seq approach, we identified both cis and trans-acting NATs in fruits. Almost all NATs (98.5%) were complementary to annotated tomato loci. Since antisense transcripts are frequently functional and play various biological roles using different transcriptional and post-transcriptional gene regulatory mechanisms in plants [49][50][51], we performed an enrichment analysis of their putative target loci. The annotation of the NAT pairs was consistent with a significant enrichment of functions related to fruit biology, such as “transporter activity” of different types of molecules. This may reflect the fact that a number of organic and inorganic molecules are transported through the plant to sink organs during fruit growth, a process that should be inhibited following fruit ripening. To our knowledge, our study is the first to reveal the occurrence and complexity of cis- and trans-NATs in tomato, also suggesting that regulation through NATs should play a role in fruits. We believe that these data will be a valuable resource for gaining insight into the complex function of the tomato transcriptome during fruit development.

### Nucleotide diversity

We also performed a comparative study of the nucleotide diversity. The RNA polarity information allowed to call variants and compare the Docet, a hybrid cultivar from a breeding company, and the Kiros, which is an inbred variety of local origin. We identified more than 17.000 single nucleotide variants compared to the reference genome, which affected around 7.000 annotated genes. Considering the intrinsic features of RNA-seq [25], the data suggested an interesting level of polymorphisms for cultivated tomato, a species which suffered severe genetic bottleneck [52]. In alfalfa, RNA-seq detected 10,826 SNPs between the two genotypes under investigation [26] while in rice, higher numbers of polymorphism were detected [25]. The Docet variety had a higher number of SNPs and polymorphic genes, and interestingly, many were heterozygous, suggesting that they are directly or indirectly related to breeding. On the other hand, the vast majority of the detected variants is not expected to have a high impact on gene function. Although limited to a reduced number of genes, the characterization of the functional consequences of variants that are predicted to have a high effect may be useful to get new insights on specific genes or functions. Taking into account the number of polymorphic site, their level of heterozygosity and the missense/nonsense ratio, this study implies that a good proportion of the polymorphisms present in the Docet cultivar should be due to breeding efforts. The data also indicated that differences in variant distribution between the two varieties were evident particularly for chromosomes 11 and 3, which also contain introgressed regions and R genes. The possibility that modern tomato breeding has increased polymorphisms, also because of the introduction of new alleles from wild species, has been already discussed and it should be tested by analyzing a larger panel of varieties [52–55].

## Conclusions

Our study provided information that may facilitate genomic analyses, allowing a more detailed description about gene function in tomato fruits. Strand specific data enabled us to identify multiple forms of non-coding RNAs that are polyadenylated, providing new insights into antisense transcripts and their potential role in gene regulation in fruits. They also allowed a more robust identification of long non-coding RNAs. Moreover, our data are useful for accurately quantifying overlapping transcripts and alternative splicing variants, although strand-specific data add time and complexity to the computational analysis and may be not always required. Finally, the annotation of the new transcripts, especially those not included in the reference genome, represents a contribution towards the complete tomato transcriptome.

The potential demonstrated by our study for the applicability of strand specific RNA-seq in gene prediction also implies that libraries covering different organs, tissues, developmental stages and a range of stress conditions are necessary to get annotations that are more comprehensive and to identify annotation-specific genes that may be important for functional studies. Given the increasing availability of genomics tools and the affordability of the NGS technologies, we hope that the here presented analysis of the tomato fruit transcriptome will be a useful resource for gene expression profile in this important organ.

## Supporting information

S1 Fig Similarity-based analysis of the TCONS transcripts. (JPEG)

S2 Fig Number of genes with at least one SNP per chromosomes. (JPEG)

S1 File Table A: GO functional enrichment analysis of the genes with splicing variants. Table B: GO functional enrichment analysis of the 1,548 tomato genes relative to the Class O transcripts. Table C: Functional annotation of the TCONS sequences. Table D: Level 2 GO terms distribution of TCONS transcripts. Table E: NAT complementarity to already annotated transcripts. Table E: Functional enrichment analysis performed on 537 tomato genes matched by 351 identified NATs.
(XLS)

## References

[1] Gnerre S, MacCallum I, Przybylski D, Ribeiro FJ, Burton JN, Walker BJ, et al High-quality draft assemblies of mammalian genomes from massively parallel sequence data. Proceedings of the National Academy of Sciences. 2011;108(4):1513–8. 10.1073/pnas.1017351108 PMC3029755 21187386

[2] Wheeler DA, Srinivasan M, Egholm M, Shen Y, Chen L, McGuire A, et al The complete genome of an individual by massively parallel DNA sequencing. Nature. 2008;452(7189):872–U5. 10.1038/nature06884 18421352

[3] Ekblom R, Galindo J. Applications of next generation sequencing in molecular ecology of non-model organisms. Heredity. 2011;107(1):1–15. 10.1038/hdy.2010.152 21139633 PMC3186121

[4] Weinstock GM. Genomic approaches to studying the human microbiota. Nature. 2012;489(7415):250–6. 10.1038/nature11553 22972298 PMC3665339

[5] Campbell PJ, Stephens PJ, Pleasance ED, O'Meara S, Li H, Santarius T, et al Identification of somatically acquired rearrangements in cancer using genome-wide massively parallel paired-end sequencing. Nat Genet. 2008;40(6):722–9. 10.1038/ng.128 18438408 PMC2705838

[6] Kim JE, Oh SK, Lee JH, Lee BM, Jo SH. Genome-Wide SNP calling using Next Generation Sequencing data in tomato. Mol Cells. 2014;37(1):36–42. 10.14348/molcells.2014.2241 24552708 PMC3907006

[7] Chen J, Kim YC, Jung YC, Xuan ZY, Dworkin G, Zhang YM, et al Scanning the human genome at kilobase resolution. Genome Res. 2008;18(5):751–62. 10.1101/gr.068304.107 18292219 PMC2336809

[8] Yeager M, Xiao NQ, Hayes RB, Bouffard P, Desany B, Burdett L, et al Comprehensive resequence analysis of a 136 kb region of human chromosome 8q24 associated with prostate and colon cancers. Hum Genet. 2008;124(2):161–70. 10.1007/s00439-008-0535-3 18704501 PMC2525844

[9] Ding L, Getz G, Wheeler DA, Mardis ER, McLellan MD, Cibulskis K, et al Somatic mutations affect key pathways in lung adenocarcinoma. Nature. 2008;455(7216):1069–75. 10.1038/nature07423 18948947 PMC2694412

[10] Mortazavi A, Williams BA, Mccue K, Schaeffer L, Wold B. Mapping and quantifying mammalian transcriptomes by RNA-Seq. Nat Methods. 2008;5(7):621–8. 10.1038/nmeth.1226 18516045 PMC13303166

[11] Marioni JC, Mason CE, Mane SM, Stephens M, Gilad Y. RNA-seq: An assessment of technical reproducibility and comparison with gene expression arrays. Genome Res. 2008;18(9):1509–17. 10.1101/gr.079558.108 18550803 PMC2527709

[12] Wang H, Chua NH, Wang XJ. Prediction of trans-antisense transcripts in Arabidopsis thaliana. Genome Biol. 2006;7(10). 10.1186/gb-2006-7-10-r92 PMC1794575 17040561

[13] Vera JC, Wheat CW, Fescemyer HW, Frilander MJ, Crawford DL, Hanski I, et al Rapid transcriptome characterization for a nonmodel organism using 454 pyrosequencing. Mol Ecol. 2008;17(7):1636–47. 10.1111/j.1365-294X.2008.03666.x 18266620

[14] Graveley BR, Brooks AN, Carlson J, Duff MO, Landolin JM, Yang L, et al The developmental transcriptome of Drosophila melanogaster. Nature. 2011;471(7339):473–9. 10.1038/nature09715 21179090 PMC3075879

[15] Trapnell C, Williams BA, Pertea G, Mortazavi A, Kwan G, van Baren MJ, et al Transcript assembly and quantification by RNA-Seq reveals unannotated transcripts and isoform switching during cell differentiation. Nat Biotechnol. 2010;28(5):511–U174. 10.1038/nbt.1621 20436464 PMC3146043

[16] Duan JL, Xia C, Zhao GY, Jia JZ, Kong XY. Optimizing de novo common wheat transcriptome assembly using short-read RNA-Seq data. Bmc Genomics. 2012;13. 10.1186/1471-2164-13-392 PMC3485621 22891638

[17] Zhang GJ, Guo GW, Hu XD, Zhang Y, Li QY, Li RQ, et al Deep RNA sequencing at single base-pair resolution reveals high complexity of the rice transcriptome. Genome Res. 2010;20(5):646–54. 10.1101/gr.100677.109 20305017 PMC2860166

[18] Roberts A, Pimentel H, Trapnell C, Pachter L. Identification of novel transcripts in annotated genomes using RNA-Seq. Bioinformatics. 2011;27(17):2325–9. 10.1093/bioinformatics/btr355 21697122

[19] Loraine AE, McCormick S, Estrada A, Patel K, Qin P. RNA-Seq of Arabidopsis pollen uncovers novel transcription and alternative splicing. Plant Physiol. 2013;162(2):1092–109. 10.1104/pp.112.211441 23590974 PMC3668042

[20] Morin RD, Bainbridge M, Fejes A, Hirst M, Krzywinski M, Pugh TJ, et al Profiling the HeLa S3 transcriptome using randomly primed cDNA and massively parallel short-read sequencing. Biotechniques. 2008;45(1):81–+. 10.2144/000112900 18611170

[21] Carninci P, Kasukawa T, Katayama S, Gough J, Frith MC, Maeda N, et al The transcriptional landscape of the mammalian genome. Science. 2005;309(5740):1559–63. 10.1126/science.1112014 16141072

[22] Nagalakshmi U, Wang Z, Waern K, Shou C, Raha D, Gerstein M, et al The transcriptional landscape of the yeast genome defined by RNA sequencing. Science. 2008;320(5881):1344–9. 10.1126/science.1158441 18451266 PMC2951732

[23] Bai Y, Dougherty L, Xu KN. Towards an improved apple reference transcriptome using RNA-seq. Mol Genet Genomics. 2014;289(3):427–38. 10.1007/s00438-014-0819-3 24532088

[24] Denoeud F, Aury JM, Da Silva C, Noel B, Rogier O, Delledonne M, et al Annotating genomes with massive-scale RNA sequencing. Genome Biol. 2008;9(12). 10.1186/gb-2008-9-12-r175 PMC2646279 19087247

[25] Lu TT, Lu GJ, Fan DL, Zhu CR, Li W, Zhao QA, et al Function annotation of the rice transcriptome at single-nucleotide resolution by RNA-seq. Genome Res. 2010;20(9):1238–49. 10.1101/gr.106120.110 20627892 PMC2928502

[26] Yang SS, Tu ZJ, Cheung F, Xu WW, Lamb JFS, Jung HJG, et al Using RNA-Seq for gene identification, polymorphism detection and transcript profiling in two alfalfa genotypes with divergent cell wall composition in stems. Bmc Genomics. 2011;12. 21504589 10.1186/1471-2164-12-199 PMC3112146

[27] Novaes E, Drost DR, Farmerie WG, Pappas GJ, Grattapaglia D, Sederoff RR, et al High-throughput gene and SNP discovery in Eucalyptus grandis, an uncharacterized genome. Bmc Genomics. 2008;9. 10.1186/1471-2164-9-312 PMC2483731 18590545

[28] Pootakham W, Shearman JR, Ruang-areerate P, Sonthirod C, Sangsrakru D, Jomchai N, et al Large-scale SNP discovery through RNA Sequencing and SNP genotyping by targeted enrichment sequencing in cassava (Manihot esculenta Crantz). Plos One. 2014;9(12). 10.1371/journal.pone.0116028 PMC4281258 25551642

[29] Liedl B, Labate JA, Stommel JR, Slade A, Kole C. Genetics, genomics and breeding of tomato. Kole, editor: CRC Press; 2013. 479 p.

[30] Tomato Genome Consortium. The tomato genome sequence provides insights into fleshy fruit evolution. Nature. 2012;485(7400):635–41. 10.1038/nature11119 22660326 PMC3378239

[31] Kumar R, Khurana A. Functional genomics of tomato: Opportunities and challenges in post-genome NGS era. J Biosciences. 2014;39(5):917–29. 10.1007/s12038-014-9480-6 25431420

[32] Martin JA, Wang Z. Next-generation transcriptome assembly. Nat Rev Genet. 2011;12(10):671–82. 10.1038/nrg3068 21897427

[33] Soil Survey Staff. Keys to Soil Taxonomy. XII ed. Washington, DC2014.

[34] Coppola V, Coppola M, Rocco M, Digilio MC, D'Ambrosio C, Renzone G, et al Transcriptomic and proteomic analysis of a compatible tomato-aphid interaction reveals a predominant salicylic acid-dependent plant response. Bmc Genomics. 2013;14. 10.1186/1471-2164-14-515 PMC3733717 23895395

[35] Bolger AM, Lohse M, Usadel B. Trimmomatic: a flexible trimmer for Illumina sequence data. Bioinformatics. 2014;30(15):2114–20. 10.1093/bioinformatics/btu170 24695404 PMC4103590

[36] Gallart AP, Pulido AH, de Lagrán IAM, Sanseverino W, Cigliano RA. GREENC: a Wiki-based database of plant lncRNAs. Nucleic Acids Res. 2016;44(D1):D1161–D6. 10.1093/nar/gkv1215 26578586 PMC4702861

[37] Kim D, Pertea G, Trapnell C, Pimentel H, Kelley R, Salzberg SL. TopHat2: accurate alignment of transcriptomes in the presence of insertions, deletions and gene fusions. Genome Biol. 2013;14(4). 10.1186/gb-2013-14-4-r36 PMC4053844 23618408

[38] Gotz S, Garcia-Gomez JM, Terol J, Williams TD, Nagaraj SH, Nueda MJ, et al High-throughput functional annotation and data mining with the Blast2GO suite. Nucleic Acids Res. 2008;36(10):3420–35. 10.1093/nar/gkn176 18445632 PMC2425479

[39] Supek F, Bošnjak M, Škunca N, Šmuc T. REVIGO summarizes and visualizes long lists of gene ontology terms. Plos One. 2011;6(7):e21800 10.1371/journal.pone.0021800 21789182 PMC3138752

[40] Argyris JM, Ruiz-Herrera A, Madriz-Masis P, Sanseverino W, Morata J, Pujol M, et al Use of targeted SNP selection for an improved anchoring of the melon (Cucumis melo L.) scaffold genome assembly. Bmc Genomics. 2015;16. 10.1186/s12864-014-1196-3 PMC4316794 25612459

[41] Cingolani P, Platts A, Wang LL, Coon M, Nguyen T, Wang L, et al A program for annotating and predicting the effects of single nucleotide polymorphisms, SnpEff: SNPs in the genome of Drosophila melanogaster strain w(1118); iso-2; iso-3. Fly. 2012;6(2):80–92. 10.4161/fly.19695 22728672 PMC3679285

[42] Jiang H, Wong WH. Statistical inferences for isoform expression in RNA-Seq. Bioinformatics. 2009;25(8):1026–32. 10.1093/bioinformatics/btp113 19244387 PMC2666817

[43] Wu PY, Phan JH, Wang MD. Assessing the impact of human genome annotation choice on RNA-seq expression estimates. Bmc Bioinformatics. 2013;14. 10.1186/1471-2105-14-S11-S8 PMC3816316 24564364

[44] Olson A, Klein RR, Dugas DV, Lu ZY, Regulski M, Klein PE, et al Expanding and vetting Sorghum bicolor gene annotations through transcriptome and methylome sequencing. Plant Genome-Us. 2014;7(2).

[45] Filichkin SA, Priest HD, Givan SA, Shen RK, Bryant DW, Fox SE, et al Genome-wide mapping of alternative splicing in Arabidopsis thaliana. Genome Res. 2010;20(1):45–58. 10.1101/gr.093302.109 19858364 PMC2798830

[46] Nilsen TW, Graveley BR. Expansion of the eukaryotic proteome by alternative splicing. Nature. 2010;463(7280):457–63. 10.1038/nature08909 20110989 PMC3443858

[47] Liu J, Jung C, Xu J, Wang H, Deng SL, Bernad L, et al Genome-Wide analysis uncovers regulation of long intergenic noncoding RNAs in Arabidopsis. Plant Cell. 2012;24(11):4333–45. 10.1105/tpc.112.102855 23136377 PMC3531837

[48] Wang J, Zhang J, Zheng H, Li J, Liu D, Li H, et al Mouse transcriptome: neutral evolution of 'non-coding' complementary DNAs. Nature. 2004;431(7010):1 p following 757; discussion following 15495343

[49] Faghihi MA, Wahlestedt C. Regulatory roles of natural antisense transcripts. Nat Rev Mol Cell Bio. 2009;10(9):637–43. 19638999 10.1038/nrm2738 PMC2850559

[50] Ietswaart R, Wu Z, Dean C. Flowering time control: another window to the connection between antisense RNA and chromatin. Trends Genet. 2012;28(9):445–53. 10.1016/j.tig.2012.06.002 22785023

[51] Pelechano V, Steinmetz LM. NON-CODING RNA Gene regulation by antisense transcription. Nat Rev Genet. 2013;14(12):880–93. 10.1038/nrg3594 24217315

[52] Aflitos S, Schijlen E, de Jong H, de Ridder D, Smit S, Finkers R, et al Exploring genetic variation in the tomato (Solanum section Lycopersicon) clade by whole-genome sequencing. Plant J. 2014;80(1):136–48. 10.1111/tpj.12616 25039268

[53] Koenig D, Jimenez-Gomez JM, Kimura S, Fulop D, Chitwood DH, Headland LR, et al Comparative transcriptomics reveals patterns of selection in domesticated and wild tomato. P Natl Acad Sci USA. 2013;110(28):E2655–E62. 10.1073/pnas.1309606110 PMC3710864 23803858

[54] Sim S-C, Van Deynze A, Stoffel K, Douches DS, Zarka D, Ganal MW, et al High-density SNP genotyping of tomato (Solanum lycopersicum L.) reveals patterns of genetic variation due to breeding. Plos One. 2012;7(9):e45520 10.1371/journal.pone.0045520 23029069 PMC3447764

[55] Corrado G, Piffanelli P, Caramante M, Coppola M, Rao R. SNP genotyping reveals genetic diversity between cultivated landraces and contemporary varieties of tomato. Bmc Genomics. 2013;14(1):1. 24279304 10.1186/1471-2164-14-835 PMC4046682

