# Supplementary figures and images for: In Silico identification and annotation of non-coding RNAs by RNA-seq and De Novo assembly of the transcriptome of Tomato Fruits
Source: PLoS One. 2017 Feb 10;12(2):e0171504. doi: 10.1371/journal.pone.0171504 (PMC5302821; doi:10.1371/journal.pone.0171504)

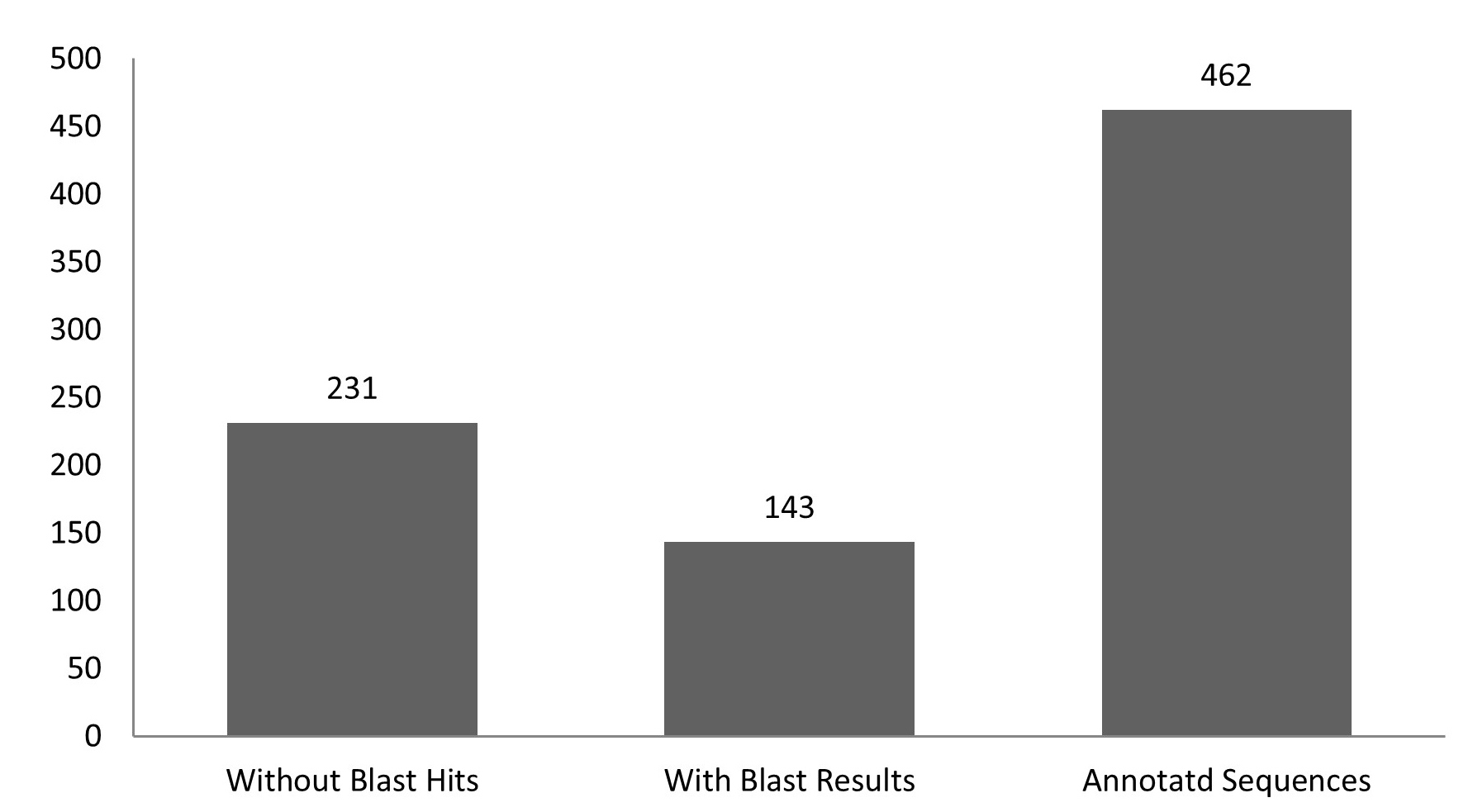

Supplement: S1 Fig — (JPEG) [file pone.0171504.s001.JPEG]

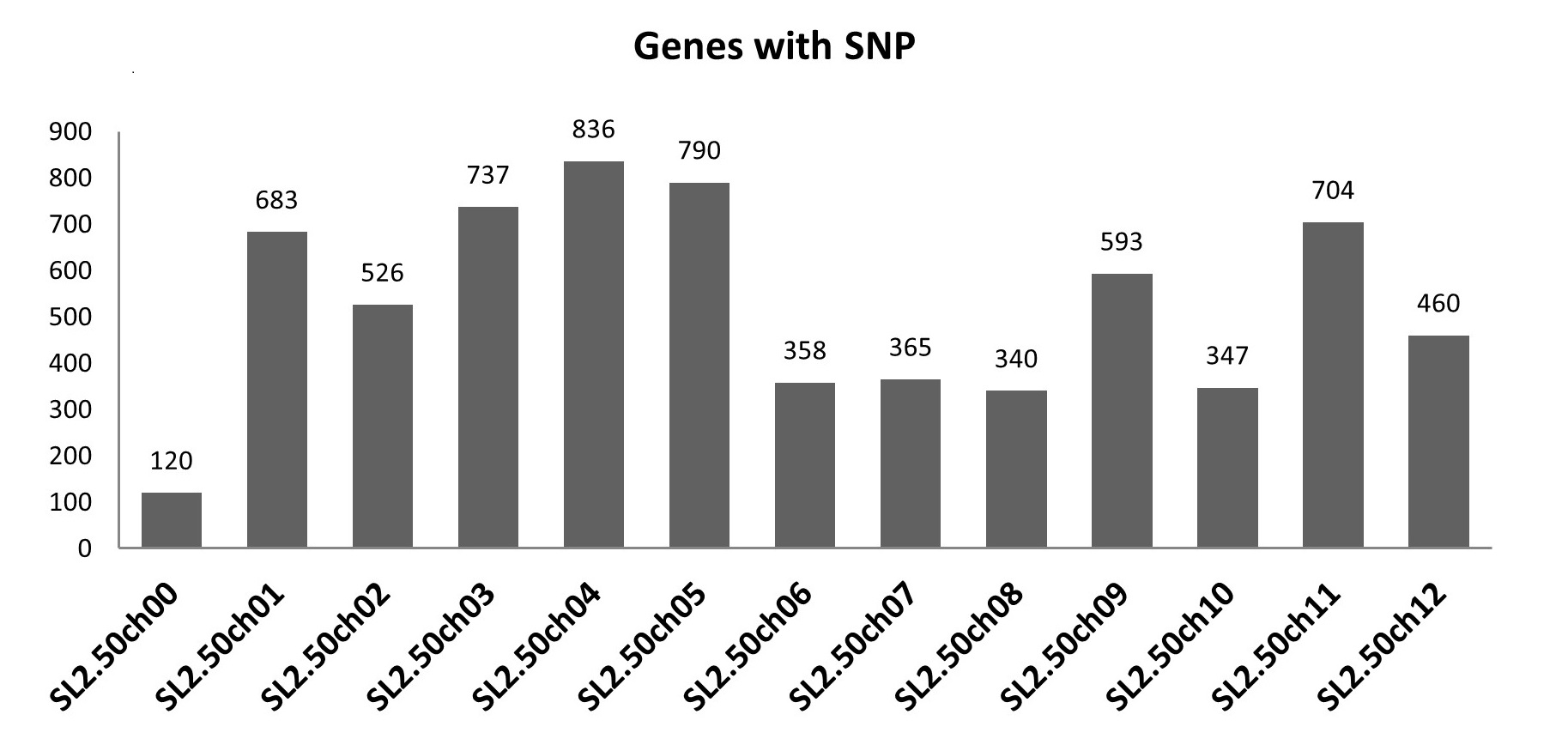

Supplement: S2 Fig — (JPEG) [file pone.0171504.s002.JPEG]
